# Coccolithophores: Functional Biodiversity, Enzymes and Bioprospecting

**DOI:** 10.3390/md9040586

**Published:** 2011-04-11

**Authors:** Emma L. Reid, Charlotte A. Worthy, Ian Probert, Sohail T. Ali, John Love, Johnathan Napier, Jenny A. Littlechild, Paul J. Somerfield, Michael J. Allen

**Affiliations:** 1 Plymouth Marine Laboratory, Prospect Place, The Hoe, Plymouth, PL1 3DH, UK; E-Mails: stal@pml.ac.uk (S.T.A.); pjso@pml.ac.uk (P.J.S.); 2 College of Life and Environmental Science, Biosciences, University of Exeter, Exeter, EX4 4QD, UK; E-Mails: e.l.reid@exeter.ac.uk (E.L.R.); j.love@exeter.ac.uk (J.L.); j.a.littlechild@exeter.ac.uk (J.A.L.); 3 Department of Biological Chemistry, Rothamsted Research, Harpenden, Herts AL5 2JQ, UK; E-Mails: charlie.worthy@bbsrc.ac.uk (C.A.W.); johnathan.napier@bbsrc.ac.uk (J.N.);; 4 CNRS-UPMC Station Biologique de Roscoff, Place Georges Teissier, 29682 Roscoff Cedex, France; E-Mail: probert@sb-roscoff.fr (I.P.)

**Keywords:** functional biodiversity, bioprospecting, biocatalysis, coccolithophore

## Abstract

*Emiliania huxleyi* is a single celled, marine phytoplankton with global distribution. As a key species for global biogeochemical cycling, a variety of strains have been amassed in various culture collections. Using a library consisting of 52 strains of *E. huxleyi* and an ‘in house’ enzyme screening program, we have assessed the functional biodiversity within this species of fundamental importance to global biogeochemical cycling, whilst at the same time determining their potential for exploitation in biocatalytic applications. Here, we describe the screening of *E. huxleyi* strains, as well as a coccolithovirus infected strain, for commercially relevant biocatalytic enzymes such as acid/alkali phosphodiesterase, acid/alkali phosphomonoesterase, EC1.1.1-type dehydrogenase, EC1.3.1-type dehydrogenase and carboxylesterase.

## 1. Introduction

Without doubt the oceanic environment represents a hotbed of microbial diversity. With an extra billion years of evolution over their terrestrial counterparts, the oceans contain some of the most ancient and diverse life forms in existence [1]. Attention was initially drawn to this potential metabolic treasure trove largely through the efforts of researchers to catalogue and assess marine biodiversity, as an academic exercise, through intense profiling of common markers such as ribosomal DNA sequence [2,3]. Yet, as our databases began to fill with newly identified permutations of well characterised marker genes, little real functional metabolic information was garnered in the process. Large scale metagenomic projects have gone some way to address this imbalance, yet relevant information on functional activity remains a sparse commodity [4,5]. This causes significant problems for both academic and applied researchers; indeed, without knowledge of the metabolic potential and activity of the individual components of complex ecosystems, the functional relevance of biodiversity remains poorly understood. This lack of understanding is particularly acute for microbial populations of similar strains which are considered as single closely-related groups with little or no attention paid to the variation contained within them which can be significant at the biochemical level.

With little functional information to hand, the first port of call for bioprospectors looking for novel metabolites, drugs and enzyme activities is often established strain libraries where the focus is often placed on screening as diverse a range of species as possible. With economics and efficiency in mind, intraspecies variation is overlooked despite the strong possibility that useful or more suitable properties may be found in “closely-related” strains to those screened. In particular, algal strains have generally been maintained within large collections, under long term continuous culture for many decades, and may therefore no longer be an accurate representation of natural activity levels, due to significant genetic drift and adaptation to artificial culture conditions.

*Emiliania huxleyi*, a single celled, lithed, marine-phytoplankton with global distribution, is the most abundant of the coccolithophores and is famous for its massive blooms which can be observed from space [6–8]. A species crucial to the study of processes including carbon and sulphur cycling in global marine systems [9], there are now over 450 known strains within culture collections around the world. Furthermore, it is host to one of the largest viruses ever discovered [10], with a genome of over 400,000 bp encoding largely novel genes [10–13]. We have assembled a diverse collection of *E. huxleyi* strains consisting of representatives established for over half a century in continuous culture as well as more recent isolates, geographically distinct strains and a virally infected strain, and assessed their biochemical diversity using a number of enzyme assays previously used to identify commercially-relevant enzyme activities from the marine environment. Enzyme activities tested for in this study were acid and alkali phosphodiesterase, acid and alkali phosphomonoesterase, EC1.1.1-type dehydrogenase, EC1.3.1-type dehydrogenase and carboxylesterase activity, respectively. Such activities could have applications in the synthesis of enantiomerically-pure chemicals for the pharmaceutical and fine chemical industry where the replacement of traditional synthetic chemistry methods is a rapidly-increasing multi-billion dollar market. We aimed to assess functional biodiversity within this species of fundamental importance to global biogeochemical cycling, whilst at the same time determining the exploitation potential of their enzymes for biocatalysis. This study demonstrates the value of screening similar strains in such biodiscovery programs.

## 2. Results and Discussion

### Enzyme Activity Assays

Fifty two strains of *Emiliania huxleyi*, isolated from various geographical locations over a period of more than half a century were acquired from ‘in-house’ and external culture collections (Table 1). All strains were screened for acid and alkali phosphodiesterase, acid and alkali phosphomonoesterase, EC1.1.1-type dehydrogenase, EC1.3.1-type dehydrogenase and carboxylesterase activity. In addition, strain CCMP2090 (a confirmed axenic strain which provides a useful ‘clean’ system for studying viral infection dynamics) was infected with the coccolithovirus EhV-86, and following harvesting 72 h later (prior to mass viral induced cellular lysis) included with the other strains. All strains displayed at least residual enzymatic activity in all the screens performed, with all tested substrates (Tables 2–10).

**Figure 1.**
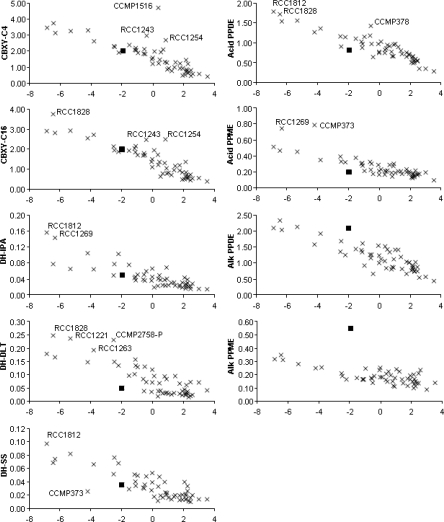
Activities of each strain for each enzyme (arbitrary units) plotted against first principle component (PC1) scores for each strain (x axis) from a PCA of normalised activities for all enzymes (×). Selected individual strains are labelled. The virally infected strain (CCMP2090inf) is indicated by ▪. Enzyme activities are carboxylesterase with C4 substrate (CBXY-C4); carboxylesterase with C16 substrate (CBXY-C16); E.C.1.1.1-type dehydrogenase with isopropyl alcohol substrate (DH-IPA); E.C.1.1.1-type dehydrogenase with DL-threonine substrate (DH-DLT); E.C.1.3.1-type dehydrogenase with sodium succinate substrate (DH-SS); alkaline phosphodiesterase (Alk PPDE); acid phosphodiesterase (Acid PPDE); alkaline phosphomonoesterase (Alk PPME); and acid phosphomonoesterase (Acid PPME).

Permutational analysis of variance based on Euclidean distances among strains showed no significant main effects of, or interaction between, strains grouped according to the sea or ocean from which they originated, or the number of years strains had been maintained in culture (Pseudo-F < 1, p > 0.7). Principal Components Analysis (PCA) showed that 69% of variation in enzyme activity among the strains could be summarised by the first principal component (PC1). All subsequent principal components had eigenvalues below 1. All enzymes had similar coefficients (range −0.365 to −0.268) on PC1. Thus despite the differences in locations from which strains were originally collected, in the lengths of time strains had been maintained in culture, and in the range of enzyme activities screened, the overall pattern was a simple gradient in overall activity (Figure 1). A strain displaying high activity in one enzyme assay tended to have high activity in the other enzyme assays.

Although 69% of the variance among strains was explained by a simple gradient in activity, strains which differ from the overall pattern in terms of the activity of one or two enzymes could be of particular interest for novel enzyme discovery and biocatalysis. To explore this possibility, actual activities of each enzyme for each strain were plotted against the scores for each strain on PC1 (Tables 2–11, Figure 1). In each plot the overall gradient from high activity to low activity is apparent. Among strains which tended to have the highest activity (the most ‘active’ 6 strains on PC1 are RCC1812, RCC1828, RCC1269, RCC1221, CCMP373, RCC1263) none had the highest activity for all enzymes. As examples, RCC1828 had high activity in the carboxylesterase screen with the C4 substrate (Table 9), while for EC1.1.1-type dehydrogenase with isopropyl alcohol substrate it was RCC1812 and RCC1269 (Table 6), for EC1.1.1-type dehydrogenase with DL-threonine substrate RCC1828, RCC1221, RCC1263 and CCMP2758 (Table 7), and so on. Even among these strains of ‘high’ overall activity some had relatively low activity for some enzymes, such as a range of strains for EC1.1.1-type dehydrogenase with isopropyl alcohol substrate (Table 6) and CCMP373 for EC1.3.1-type dehydrogenase (Table 7). Some strains which generally had mid-range activity for most enzymes (*i.e.*, have PC1 scores between −2 and +2) had relatively high activity for individual enzymes (Figure 1), such as CCMP1516 for carboxylesterase activity with C4 substrate; RCC1243 and RCC1254 for carboxylesterase activity with both C4 and C16 substrates; and CCMP378 for acid phosphodiesterase activity.

Among the strains screened are some that might be expected to be highly similar in terms of their enzyme activity. CCMP373 and CCMP88E are thought to be the same strain, but they clearly differ in terms of their activity, as CCMP373 is identified as having relatively high overall activity (low PC1 score) with low dehydrogenase activity (sodium succinate substrate) and high acid phosphomonoesterase activity (Figure 1). Likewise CCMP2090 and CCMP1516 are also thought to be synonyms, but CCMP1516 is identified as having high activity for carboxylesterase (C4 substrate) whereas CCMP2090 is not. Although synonyms, CCMP2090 is an axenic version of CCMP1516 which fails to calcify. The physiological differences between CCMP1516 and CCMP2090 may account for the difference in carboxylesterase activity displayed. CCMP2758-P and CCMP2758-B are definitely the same strain, cultured separately in different collections for approximately 7 years, yet CCMP2758-P displayed a higher overall activity (lower PC1 score), especially in the dehydrogenase assay (DL-threonine substrate) (Figure 1). CCMP376-B and CCMP376-P, also cultured separately for 7 years, display no evidence of differences in activity. Moreover, despite the significant changes in cellular physiology between the haploid (motile) and diploid (lithed) state in *E. huxleyi*, RCC1217 and RCC1216 (haploid and diploid manifestations of the same strain) displayed no significant evidence of differences in activity in the assays tested. Previous studies have shown significant overlap (approximately 50%) exists between the transcriptional profiles of RCC1216 and RCC1217 with a core set of 3,519 EST clusters identified as common to both life stages [14]. Furthermore, 22 of these EST clusters display database homology to known esterases (including phosphomonoesterases, phosphodiesterases and carboxylesterases), while 94 display homology to known dehydrogenases (including succinate and threonine dehydrogenases) (see supplementary material of [14]). That is not to say that further investigation will not reveal significant metabolic differences between RCC1217 and RCC1216, however. These limited examples raise several crucial issues for further research, such as the repeatability of screening results, the reliability of strain-identification methods, and the relationships between function and taxonomy.

Of particular note is the difference in alkaline phosphomonoesterase and phosphodiesterase activity displayed by the EhV-86 infected strain of CCMP2090 in comparison with the uninfected CCMP2090, and other *E. huxleyi* strains. With a PC1 score of 1.93 for CCMP2090 and −1.96 for CCMP2090inf, the infected strain generally displayed higher overall activities in all enzyme assays than its uninfected counterpart. The reason for this is, as yet, unclear, but could be a physical effect of the infection process (e.g., variation in cellular integrity or segregation) or a biochemical effect (e.g., variation in metabolism). The infected strain, CCMP2090inf, is highlighted in each plot in Figure 1. Viral infection had little effect on relative carboxylesterase activity (with either C4 or C16 substrate); reduced E.C.1.1.1-type dehydrogenase with isopropyl alcohol substrate and E.C.1.3.1-type dehydrogenase activity slightly; reduced E.C.1.1.1-type dehydrogenase with DL-threonine substrate markedly; and reduced acid phosphodiesterase and phosphomonoesterase activity. However, viral infection had the effect of increasing both alkaline phosphomonoesterase and phosphodiesterase activity, especially the former. Indeed, EhV-86 infected CCMP2090 displayed a higher alkaline phosphomonoesterase activity than all the tested strains of *E. huxleyi.*

The higher activity observed in this assay may be due to the upregulation or increased activity of *E. huxleyi* phosphonomonoesterase function in response to viral infection. However, the increased activity could also be a direct consequence of infection through the action of virally encoded enzymes. Indeed, the EhV-86 genome has revealed two such candidates (ehv028 and ehv363) for this activity in the form of coding sequences which have homology to known esterases [10]. Whilst transcripts for ehv363 have so far not been detected during global transcriptional analysis of the infection cycle, transcripts for ehv028 have been detected within two hours of infection by EhV-86 [15].

## 3. Experimental Section

### 3.1. Strain Culture and Harvesting

The strains used in this study are shown in Table 1. For each strain of *E. huxleyi*, 500 mL of F/2 (Guillard 1975) was seeded with 25 mL of mid exponential starter culture [16]. The cultures were grown at 15 °C with a photoperiod of 16 h:8 h L:D. Culture flasks were gently shaken once per day until mid-exponential growth (4 × 10^6^ cells mL^−1^) was reached. Biomass was harvested by centrifugation at 8000 g for 30 min at 15 °C. CCMP2090-B was infected 72 h prior to harvesting with 0.5 mL Emiliania huxleyi Virus 86 (EhV-86) giving MOI of 1:1.

### 3.2. Enzyme Activity Assays

Cell pellets were resuspended in 2.5 mL of 50 mM potassium phosphate buffer (pH 7.0) containing 5 mg/mL polyethylenimine and disrupted by sonication on ice. Cell debris was removed by centrifugation at 4,000 g for 10 mins at 4 °C and the protein concentration of extracts determined using Bradford’s assay. Enzyme assays were carried out in triplicate in 96 well, flat bottom microplates using 50 μL of cell extract per reaction in a total assay volume of 250 μL. Reaction mixes were incubated at room temperature for 60 min and absorbance changes (due to colour development) were monitored using a Molecular Devices Versamax platereader at 415 nm.

Acid or alkaline phosphodiesterase activity was measured by incubating extract plus bis-(4-nitrophenyl) phosphate (20 mM) in the presence of either 11.5 mM HCl or 7mM NaOH, respectively. Similarly, for acid or alkali phosphomonoesterase activity, extract plus 4-nitrophenyl phosphate (20 mM) was incubated with either 11.5 mM HCl or 7 mM NaOH, respectively. Carboxylesterase activity was detected by incubating extract in the presence of either 4-nitrophenyl butyrate (C4) or 4-nitrophenyl palmitate (C16) at a final concentration of 20 mM, respectively. EC.1.1.1-type dehydrogenase activity was detected incubating extract as follows: isopropyl alcohol or DL-threonine (20 mM), NaOH (7 mM), NAD (1 mM), XTT (0.5 mM), and 10.25 Units of Diaphorase solution. EC.1.3.1-type dehydrogenase activity was detected in an identical assay mix except that sodium succinate (20 mM) replaced the isopropyl alcohol or DL-threonine as substrate.

### 3.3. Statistical Analysis

Data were normalised to protein content. To account for differences in average activity among enzymes data were standardised across all strains by subtracting the mean activity and dividing through by the standard deviation. This placed the variation in activity for each enzyme across all strains on a scale of standard deviations centered on zero. The standardised dataset was analysed using multivariate methods in Primer v6 [17,18] with the Permanova+ add-in [19].

## 4. Conclusions

All *E. huxleyi* strains under study displayed acid and alkali phosphodiesterase, acid and alkali phosphomonoesterase, EC1.1.1-type dehydrogenase, EC1.3.1-type dehydrogenase and carboxylesterase activity with all variants of the substrates tested. Strains displaying higher activities for one enzyme function tended also to have higher activities for the other enzyme functions tested. Consequently, we observed a simple gradient in enzyme activity, from low activity strains to high activity strains. Along this gradient, we identified six strains displaying significantly higher enzymatic activities than their relatives. On the whole, strains of *E. huxleyi* displayed similar metabolic potentials, yet variations did occur within some strains which exhibited marked increases or decreases in particular enzyme activities relative to their “expected” activity (*i.e.*, the gradual changes in enzymatic activity observed in the general population). These variations could have profound effects on ecosystem productivity and form the basis of functional biodiversity. Crucially, the activity gradient was skewed only on a few occasions, notably by viral infection. The display of increased phosphomonoesterase activity in virally infected cells is a particularly noteworthy example of this departure from the norm. As arguably the largest reservoir of genetic novelty on the planet, the metabolic potential of viruses is enormous. As we have shown here, viruses have much to offer the field of biocatalysis. Moreover, with their relatively small genomes, gene identification is not as arduous a task as it can be with the larger genomes found within their hosts. However, despite the massive potential for viruses in biocatalysis, the problem of identifying suitable hosts for culture-dependent enzyme screening of the nature undertaken in this study remains significant. Of further interest to biodiscovery programs, enzyme activity was not associated with geographic location or the length of time strains had been in culture, suggesting that, for preliminary screens, established culture collections are indeed a useful and valid starting point. A high degree of genetic diversity has previously been observed among *E. huxleyi* strains [20], as well as for other algal species [21], yet the ecological and functional relevance of this diversity has so far remained unassessed. The results here demonstrate that once a specific enzyme functional activity is identified in any particular strain under study, the screening of related strains (both close and distant relatives) for altered activity levels is a prudent and worthwhile approach for both ecological and biotechnological applications.

## Figures and Tables

**Table 1.** Strains of *Emiliania huxleyi* used in this study.

**Strain**	**Source**	**Date**	**Strain**	**Source**	**Date**
*CCMP2090	Pacific Ocean—Ecuadorian Coast	1991	RCC1812	Mediterranean Sea	2008
CCMP12.1	Atlantic Ocean—Sargasso Sea	1987	RCC1818	Mediterranean Sea	2008
#CCMP88E	Atlantic Ocean—Sargasso Sea	1960	RCC1826	Mediterranean Sea	2008
CCMP370	Atlantic Ocean—North Sea	1959	RCC1828	Mediterranean Sea	2008
CCMP372	Atlantic Ocean—Sargasso Sea	1987	RCC1830	Mediterranean Sea	2008
#CCMP373	Atlantic Ocean—Sargasso Sea	1960	RCC1850	Mediterranean Sea	2008
CCMP374	Atlantic Ocean—Gulf of Maine	1989	RCC2054	Mediterranean Sea	2008
CCMP376-P	Atlantic Ocean—Gulf of Maine	1986	RCC1269	Atlantic Ocean	2007
CCMP376-B	Atlantic Ocean—Gulf of Maine	1986	RCC1268	Atlantic Ocean	2007
CCMP378	Atlantic Ocean—Gulf of Maine	1988	RCC1270	Atlantic Ocean	2007
CCMP379	English Channel	1957	RCC1267	Atlantic Ocean	2007
CCMP625	Not known	2006	RCC912	Pacific Ocean—Marquises islands	2004
*CCMP1516	Pacific Ocean—Ecuadorian Coast	1991	RCC948	Pacific Ocean—South East Pacific	2004
CCMP2758-P	Pacific Ocean—Gulf of Alaska	2006	RCC958	Pacific Ocean—Marquises Islands	2004
CCMP2758-B	Pacific Ocean—Gulf of Alaska	2006	RCC962	Pacific Ocean—Marquises Islands	2004
RCC1263	Atlantic Ocean—Ireland	2007	RCC1261	Mediterranean Sea—Spanish coast	1999
RCC1271	Atlantic Ocean—Ireland	2007	RCC1246	Mediterranean Sea—Spanish coast	1999
RCC1250	Mediterranean Sea—Alboran Sea	1999	RCC1257	Atlantic Ocean—Icelandic coast	1991
RCC1221	Mediterranean Sea—Alboran Sea	1999	RCC1256	Atlantic Ocean—Icelandic coast	1991
RCC1254	Mediterranean Sea—Alboran Sea	1999	PLY92A	English Channel	1957
RCC1208	Mediterranean Sea—Alboran Sea	1999	RCC1222	Baltic Sea—Swedish coast	1998
RCC1248	Atlantic Ocean—Portugal	1999	BLOOM2195	English Channel	1999
RCC1251	Atlantic Ocean—Portugal	1999	RCC1258	Atlantic Ocean—Ireland	1998
RCC1710	Japan	2007	CH24/90	Indian Ocean—NZ Coast	1992
RCC1217	Pacific Ocean—Tasman Sea	1998	5-9-25B	North Atlantic	1990
RCC1216	Pacific Ocean—Tasman Sea	1998	RCC1243	Northern Spain	2002

* CCMP2090/CCMP1516 and

# CCMP88E/CCMP373 are pseudonyms of the same strains.

The B suffix denotes a strain obtained from Bigelow (CCMP) directly prior to this study, a P suffix denotes a strain from Bigelow (CCMP) already in culture at PML prior to this study.

**Table 2.** Acid phosphomonoesterase (PPME) activity displayed by various *E. huxleyi* strains (arbitrary values).

**Strain**	**Acid PPME**		**Strain**	**Acid PPME**	
	**Activity**	**St Dev**		**Activity**	**St Dev**
CCMP2090	0.17961	0.01539	RCC1812	0.51630	0.10917
CCMP2090inf	0.19424	0.00928	RCC1818	0.17238	0.00087
CCMP1516	0.16531	0.00290	RCC1826	0.20055	0.00458
CCMP 12-1	0.17563	0.00844	RCC1828	0.46822	0.00322
CCMP88E	0.13792	0.00339	RCC1830	0.31137	0.04407
**CCMP373**	**0.78789**	**0.04376**	RCC1850	0.15542	0.00791
CCMP370	0.19339	0.00631	RCC2054	0.18864	0.00347
CCMP372	0.13417	0.01233	**RCC1269**	**0.74327**	**0.15210**
CCMP374	0.16857	0.00709	RCC1268	0.27304	0.05692
CCMP376-P	0.37632	0.02432	RCC1270	0.32470	0.04458
CCMP376-B	0.23170	0.00434	RCC1267	0.16693	0.00359
CCMP378	0.22527	0.00329	RCC912	0.17509	0.00945
CCMP379	0.21367	0.00578	RCC948	0.23107	0.00760
CCMP625	0.32951	0.06536	RCC958	0.32239	0.08688
CCMP2758-P	0.38968	0.09136	RCC962	0.14034	0.00297
CCMP2758-B	0.21094	0.00124	RCC1261	0.18559	0.00133
RCC1263	0.34975	0.01739	RCC1246	0.30096	0.01448
RCC1271	0.30134	0.00911	RCC1257	0.21443	0.00780
RCC1250	0.20014	0.01012	RCC1256	0.20445	0.00779
RCC1221	0.45293	0.01948	PLY92A	0.21706	0.00958
RCC1254	0.14088	0.00192	RCC1222	0.20877	0.00179
RCC1208	0.16183	0.00392	BLOOM2195	0.12373	0.05572
RCC1248	0.21912	0.02420	RCC1258	0.19028	0.00276
RCC1251	0.27738	0.00216	CH24/90	0.28767	0.02433
RCC1710	0.30138	0.08715	5-9-25B	0.20566	0.00246
RCC1217	0.09583	0.00125	RCC1243	0.17680	0.00478
RCC1216	0.28645	0.01523			

Entries in bold denote strains displaying high activities.

**Table 3.** Alkali phosphomonoesterase (PPME) activity displayed by various *E. huxleyi* strains (arbitrary values).

**Strain**	**Alkali PPME**		**Strain**	**Alkali PPME**	
	**Activity**	**Std Dev**		**Activity**	**Std Dev**
CCMP2090	0.19366	0.00951	RCC1812	0.31577	0.02209
**CCMP2090inf**	**0.55219**	**0.01506**	RCC1818	0.13591	0.00566
CCMP1516	0.16258	0.01049	RCC1826	0.14854	0.00502
CCMP12-1	0.23399	0.05954	**RCC1828**	**0.34873**	**0.00139**
CCMP88E	0.14955	0.00922	RCC1830	0.16449	0.00537
CCMP373	0.25032	0.01367	RCC1850	0.11324	0.00895
CCMP370	0.08650	0.00507	RCC2054	0.15019	0.03608
CCMP372	0.14826	0.04771	RCC1269	0.31049	0.01740
CCMP374	0.12796	0.00980	RCC1268	0.11152	0.00623
CCMP376-P	0.17664	0.00666	RCC1270	0.17496	0.01980
CCMP376-B	0.11524	0.00550	RCC1267	0.18210	0.00519
CCMP378	0.16864	0.04599	RCC912	0.20918	0.03438
CCMP379	0.20780	0.00538	RCC948	0.18221	0.00421
CCMP 625	0.19062	0.01509	RCC958	0.16516	0.01366
CCMP2758-P	0.20995	0.00784	RCC962	0.10016	0.00814
CCMP2758-B	0.24732	0.00857	RCC1261	0.16676	0.00926
RCC1263	0.25610	0.01770	RCC1246	0.17552	0.00798
RCC1271	0.16352	0.01319	RCC1257	0.25125	0.02641
RCC1250	0.16956	0.01280	RCC1256	0.16433	0.00759
RCC1221	0.28018	0.00894	PLY92A	0.22421	0.00354
RCC1254	0.11557	0.00349	RCC1222	0.16387	0.00372
RCC1208	0.13661	0.01011	BLOOM2195	0.20202	0.03559
RCC1248	0.13519	0.02426	RCC1258	0.23966	0.04469
RCC1251	0.16360	0.01027	CH24/90	0.23484	0.00194
RCC1710	0.14002	0.00485	5-9-25B	0.22395	0.00937
RCC1217	0.13866	0.01339	RCC1243	0.18878	0.00606
RCC1216	0.19610	0.00894			

Entries in bold denote strains displaying high activities.

**Table 4.** Acid phosphodiesterase (PPDE) activity displayed by various *E. huxleyi* strains (arbitrary values).

**Strain**	**Acid PPDE**		**Strain**	**Acid PPDE**	
	**Activity**	**Std Dev**		**Activity**	**Std Dev**
CCMP2090	0.71535	0.03780	**RCC1812**	**1.78429**	**0.26475**
CCMP2090inf	0.81663	0.01473	RCC1818	0.71560	0.02312
CCMP1516-P	0.74463	0.05355	RCC1826	0.85526	0.02156
CCMP12-1	0.34747	0.04737	**RCC1828**	**1.71480**	**0.08981**
CCMP88E	0.55860	0.02616	RCC1830	0.88175	0.02302
CCMP373	1.26092	0.08947	RCC1850	0.51413	0.05150
CCMP370	0.34545	0.03623	RCC2054	0.77038	0.01277
CCMP372	0.50588	0.03153	**RCC1269**	**1.53905**	**0.11675**
CCMP374	0.65942	0.03761	RCC1268	0.66338	0.03980
CCMP376-P	1.10460	0.10275	RCC1270	1.17517	0.12956
CCMP376-B	0.64738	0.06432	RCC1267	0.77207	0.03226
CCMP378	1.42860	0.00885	RCC912	0.53782	0.02768
CCMP379	0.81666	0.01853	RCC948	1.03075	0.08816
CCMP625	0.90846	0.14299	RCC958	1.14167	0.10030
CCMP2758-P	1.15915	0.13235	RCC962	0.64502	0.01470
CCMP2758-B	1.15550	0.08619	RCC1261	0.48601	0.02034
RCC1263	1.35473	0.11517	RCC1246	0.94996	0.07626
RCC1271	1.02994	0.06474	RCC1257	0.74399	0.04046
RCC1250	0.75125	0.09794	RCC1256	0.69247	0.04739
**RCC1221**	**1.55556**	**0.26597**	PLY92A	0.94201	0.10085
RCC1254	0.66117	0.01171	RCC1222	0.80130	0.01172
RCC1208	0.55242	0.06229	BLOOM2195	0.59083	0.01506
RCC1248	0.59579	0.04379	RCC1258	0.97228	0.05713
RCC1251	0.97180	0.09077	CH24/90	0.97041	0.02280
RCC1710	0.97134	0.05270	5-9-25B	0.89314	0.05387
RCC1217	0.28298	0.03174	RCC1243	0.64056	0.01086
RCC1216	0.91697	0.12004			

Entries in bold denote strains displaying high activities.

**Table 5.** Alkali phosphodiesterase (PPDE) activity displayed by various *E. huxleyi* strains (arbitrary values).

**Strain**	**Alkali PPDE**		**Strain**	**Alkali PPDE**	
	**Activity**	**Std Dev**		**Activity**	**Std Dev**
CCMP2090	1.01498	0.03284	**RCC1812**	**2.09800**	**0.16065**
**CCMP2090inf**	**2.09356**	**0.09370**	RCC1818	0.81822	0.00583
CCMP1516	1.06632	0.16400	RCC1826	0.83726	0.01113
CCMP12-1	0.54110	0.01648	**RCC1828**	**2.34065**	**0.35407**
CCMP88E	0.73552	0.06820	RCC1830	1.34868	0.19322
CCMP373	1.57758	0.07356	RCC1850	0.74402	0.00866
CCMP370	0.56268	0.02989	RCC2054	0.79718	0.00362
CCMP372	0.77211	0.03632	RCC1269	2.03660	0.28402
CCMP374	0.88076	0.00574	RCC1268	0.85921	0.00252
CCMP376-P	1.43431	0.03418	RCC1270	1.26434	0.07075
CCMP376-B	0.65800	0.05355	RCC1267	0.99680	0.09117
CCMP378	1.15499	0.05097	RCC912	0.87723	0.08615
CCMP379	1.52023	0.17445	RCC948	1.11699	0.02386
CCMP625	1.68249	0.02327	RCC958	1.25341	0.04583
CCMP2758-P	1.35426	0.17741	RCC962	0.84704	0.01028
CCMP2758-B	0.91871	0.09512	RCC1261	0.80675	0.03748
RCC1263	1.92140	0.04877	RCC1246	1.12463	0.13606
RCC1271	1.40183	0.16875	RCC1257	1.24291	0.05902
RCC1250	1.03431	0.00522	RCC1256	1.01402	0.05034
**RCC1221**	**2.13127**	**0.11071**	PLY92A	1.42313	0.00162
RCC1254	0.86972	0.00557	RCC1222	1.18854	0.01239
RCC1208	0.74311	0.02046	BLOOM2195	0.93662	0.04273
RCC1248	1.02170	0.02901	RCC1258	1.40924	0.03235
RCC1251	1.61663	0.04860	CH24/90	1.53915	0.09025
RCC1710	1.26281	0.16583	5-9-25B	1.40952	0.03855
RCC1217	0.43044	0.01153	RCC1243	0.65717	0.02023
RCC1216	1.34065	0.05008			

Entries in bold denote strains displaying high activities.

**Table 6.** E.C.1.1.1-type dehydrogenase activity (isopropyl alcohol substrate, DH-IPA) displayed by various *E. huxleyi* strains (arbitrary values).

**Strain**	**DH-IPA**		**Strain**	**DH-IPA**	
	**Activity**	**Std Dev**		**Activity**	**Std Dev**
CCMP2090	0.01859	0.00091	**RCC1812**	**0.15706**	**0.01907**
CCMP2090inf	0.04983	0.00080	RCC1818	0.02373	0.00577
CCMP1516	0.02230	0.00586	RCC1826	0.04896	0.00194
CCMP12-1	0.02799	0.00502	RCC1828	0.07718	0.02393
CCMP88E	0.02996	0.00247	RCC1830	0.04540	0.01346
**CCMP373**	**0.10472**	**0.03671**	RCC1850	0.03843	0.00227
CCMP370	0.01530	0.00907	RCC2054	0.03811	0.00333
CCMP372	0.04377	0.00297	**RCC1269**	**0.14375**	**0.01265**
CCMP374	0.02062	0.00308	RCC1268	0.06893	0.01351
CCMP376-P	0.07574	0.01807	RCC1270	0.03873	0.00103
CCMP376-B	0.02102	0.00300	RCC1267	0.06616	0.00180
CCMP378	0.05063	0.00438	RCC912	0.02083	0.00207
CCMP379	0.04085	0.01177	RCC948	0.01846	0.00474
CCMP625	0.07719	0.00417	RCC958	0.10264	0.00864
CCMP2758	0.04864	0.00951	RCC962	0.01629	0.00182
CCMP2758-B	0.05074	0.00287	RCC1261	0.02589	0.02280
RCC1263	0.06434	0.00928	RCC1246	0.04088	0.00037
RCC1271	0.04228	0.00997	RCC1257	0.04499	0.00471
RCC1250	0.02493	0.00371	RCC1256	0.03144	0.01738
RCC1221	0.06522	0.01793	PLY92A	0.03740	0.00804
RCC1254	0.02331	0.00261	RCC1222	0.03121	0.00718
RCC1208	0.01528	0.00846	BLOOM2195	0.02737	0.00617
RCC1248	0.02063	0.00227	RCC1258	0.02449	0.00184
RCC1251	0.03713	0.01565	CH24/90	0.03571	0.00404
RCC1710	0.03576	0.01327	5-9-25B	0.03008	0.00745
RCC1217	0.01462	0.00462	RCC1243	0.05970	0.00298
RCC1216	0.02415	0.00335			

Entries in bold denote strains displaying high activities.

**Table 7.** E.C.1.1.1-type dehydrogenase activity (DL-threonine substrate, DH-DLT) displayed by various *E. huxleyi* strains (arbitrary values).

**Strain**	**DH-DLT**		**Strain**	**DH-DLT**	
	**Activity**	**Std Dev**		**Activity**	**Std Dev**
CCMP2090	0.02210	0.00242	RCC1812	0.17880	0.01746
CCMP2090inf	0.05144	0.00607	RCC1818	0.09427	0.03561
CCMP1516	0.01792	0.00440	RCC1826	0.06735	0.01943
CCMP12-1	0.02436	0.00624	**RCC1828**	**0.24829**	**0.03027**
CCMP88E	0.02772	0.00255	RCC1830	0.15705	0.00788
CCMP373	0.14775	0.02680	RCC1850	0.03672	0.00079
CCMP370	0.07007	0.00911	RCC2054	0.09311	0.00333
CCMP372	0.04402	0.00278	RCC1269	0.16521	0.01267
CCMP374	0.03151	0.00534	RCC1268	0.08357	0.01715
CCMP376-P	0.09640	0.00586	RCC1270	0.08399	0.00768
CCMP376-B	0.06679	0.00179	RCC1267	0.07144	0.00497
CCMP378	0.03853	0.00314	RCC912	0.01955	0.00395
CCMP379	0.03778	0.00819	RCC948	0.09411	0.01239
CCMP625	0.14808	0.01738	RCC958	0.13437	0.01311
CCMP2758-P	0.23046	0.01668	RCC962	0.02285	0.00151
**CCMP2758-B**	**0.12875**	**0.00213**	RCC1261	0.06040	0.00638
RCC1263	0.19303	0.01293	RCC1246	0.03275	0.00741
RCC1271	0.10255	0.02299	RCC1257	0.11920	0.00962
RCC1250	0.02776	0.00481	RCC1256	0.03870	0.01396
RCC1221	0.23650	0.02854	PLY92A	0.04026	0.00832
RCC1254	0.03170	0.00137	RCC1222	0.03048	0.00777
RCC1208	0.07265	0.00819	BLOOM2195	0.02652	0.00642
RCC1248	0.01594	0.00079	RCC1258	0.02770	0.00434
RCC1251	0.13390	0.02492	CH24/90	0.03547	0.00404
RCC1710	0.13055	0.01102	5-9-25B	0.02561	0.00521
RCC1217	0.04174	0.00496	RCC1243	0.07169	0.00182
RCC1216	0.02934	0.00622			

Entries in bold denote strains displaying high activities.

**Table 8.** E.C.1.3.1-type dehydrogenase activity (sodium succinate substrate, DH-SS) displayed by various *E. huxleyi* strains (arbitrary values).

**Strain**	**DH-SS**		**Strain**	**DH-SS**	
	**Activity**	**Std Dev**		**Activity**	**Std Dev**
CCMP2090	0.01303	0.00208	**RCC1812**	**0.09719**	**0.00000**
CCMP2090inf	0.03563	0.00764	RCC1818	0.01991	0.00494
CCMP1516-P	0.01135	0.00367	RCC1826	0.04740	0.00654
CCMP12-1	0.01994	0.00437	RCC1828	0.06835	0.02432
CCMP88E	0.02584	0.00235	RCC1830	0.04044	0.00549
CCMP373	0.02546	0.02951	RCC1850	0.03343	0.00090
CCMP370	0.01377	0.01196	RCC2054	0.03984	0.00770
CCMP372	0.03945	0.00372	RCC1269	0.07398	0.01069
CCMP374	0.01947	0.00256	RCC1268	0.02026	0.00615
CCMP376-P	0.02860	0.02781	RCC1270	0.03130	0.01443
CCMP376-B	0.01931	0.00043	RCC1267	0.05337	0.00960
CCMP378	0.03019	0.00419	RCC912	0.01583	0.00179
CCMP379-B	0.03268	0.00117	RCC948	0.01756	0.00844
**CCMP625**	**0.07641**	**0.00984**	RCC958	0.06765	0.00570
CCMP2758-P	0.05166	0.01193	RCC962	0.01314	0.00038
CCMP2758-B	0.04932	0.00321	RCC1261	0.01409	0.00426
RCC1263	0.06626	0.01356	RCC1246	0.02333	0.00499
RCC1271	0.05092	0.01477	RCC1257	0.03934	0.00326
RCC1250	0.01273	0.00883	RCC1256	0.02063	0.01396
**RCC1221**	**0.08224**	**0.01251**	PLY92A	0.01921	0.00614
RCC1254	0.02360	0.00107	RCC1222	0.01734	0.00767
RCC1208	0.00941	0.00374	BLOOM2195	0.02130	0.00730
RCC1248	0.01078	0.00090	RCC1258	0.01566	0.00276
RCC1251	0.03377	0.01087	CH24/90	0.02691	0.00377
RCC1710	0.03576	0.01023	5-9-25B	0.01382	0.00153
RCC1217	0.01356	0.00316	RCC1243	0.04835	0.00912
RCC1216	0.01664	0.00326			

Entries in bold denote strains displaying high activities.

**Table 9.** Carboxylesterase activity (C4 substrate, CBXY-C4) displayed by various *E. huxleyi* strains (arbitrary values).

**Strain**	**CBXY-C4**		**Strain**	**CBXY-C4**	
	**Activity**	**Std Dev**		**Activity**	**Std Dev**
CCMP2090	0.91263	0.05811	RCC1812	3.47836	0.22679
CCMP2090inf	2.01264	0.03272	RCC1818	1.28586	0.06262
**CCMP1516**	**4.72720**	**0.08391**	RCC1826	1.46971	0.01877
CCMP12-1	0.56503	0.01607	**RCC1828**	**3.75741**	**0.04434**
CCMP88E	1.19434	0.02193	RCC1830	2.19248	0.08135
CCMP373	3.30686	0.02323	RCC1850	0.60506	0.00868
CCMP370	0.57237	0.00195	RCC2054	1.31421	0.07909
CCMP372	0.52416	0.01124	RCC1269	3.14825	0.06077
CCMP374	0.85715	0.05896	RCC1268	1.26878	0.07059
CCMP376-P	2.37180	0.12357	RCC1270	1.83042	0.02733
CCMP376-B	1.23213	0.11012	RCC1267	1.57030	0.13579
CCMP378	2.12765	0.05996	RCC912	0.44616	0.01026
CCMP379	1.22888	0.07401	RCC948	1.98188	0.05798
CCMP625	2.27299	0.11844	RCC958	1.92105	0.04765
CCMP2758-P	2.31912	0.15994	RCC962	1.44293	0.03853
CCMP2758-B	1.87580	0.10302	RCC1261	0.76397	0.01772
RCC1263	2.61899	0.06146	RCC1246	0.84784	0.01731
RCC1271	2.07483	0.06317	RCC1257	1.20087	0.02386
RCC1250	0.90858	0.07761	RCC1256	0.46525	0.02618
RCC1221	3.24845	0.07028	PLY92A	1.92798	0.06885
RCC1254	2.69194	0.05944	RCC1222	0.99775	0.15048
RCC1208	0.80126	0.01865	BLOOM2195	0.69953	0.01288
RCC1248	0.71100	0.02739	RCC1258	0.93082	0.01764
RCC1251	2.05211	0.07192	CH24/90	1.48482	0.02644
RCC1710	1.80632	0.09925	5-9-25B	0.74347	0.02828
RCC1217	0.41034	0.01356	RCC1243	2.96084	0.19796
RCC1216	2.09708	0.02705			

Entries in bold denote strains displaying high activities.

**Table 10.** Carboxylesterase activity (C16 substrate, CBXY-C16) displayed by various *E. huxleyi* strains (arbitrary values).

**Strain**	**CBXY-C16**		**Strain**	**CBXY-C16**	
	**Activity**	**Std Dev**		**Activity**	**Std Dev**
CCMP2090	0.83590	0.03418	RCC1812	2.90956	0.17719
CCMP2090inf	1.93762	0.16983	RCC1818	1.14399	0.00598
CCMP1516	1.71990	0.03231	RCC1826	1.35472	0.11735
CCMP12-1	0.53526	0.05624	**RCC1828**	**3.75648**	**0.18971**
CCMP88E	0.50175	0.04024	RCC1830	1.85034	0.00488
CCMP373	2.54264	0.07732	RCC1850	0.59257	0.09620
CCMP370	0.55533	0.02635	RCC2054	1.30630	0.01170
CCMP372	0.48642	0.00696	RCC1269	2.80862	0.20754
CCMP374	0.82781	0.07642	RCC1268	1.04188	0.07728
CCMP376-P	1.91921	0.09312	RCC1270	1.68226	0.12782
CCMP376-B	1.19587	0.03814	RCC1267	1.49031	0.12209
CCMP378	1.79742	0.11825	RCC912	0.43431	0.01801
CCMP379	1.21532	0.12853	RCC948	1.93536	0.04057
CCMP625	1.93508	0.07774	RCC958	1.86366	0.31931
CCMP2758-P	2.12730	0.12864	RCC962	1.36145	0.09054
CCMP2758-B	1.70422	0.04218	RCC1261	0.70427	0.04096
RCC1263	2.72156	0.42697	RCC1246	0.77306	0.05014
RCC1271	2.04049	0.06138	RCC1257	1.09724	0.14293
RCC1250	0.92810	0.06444	RCC1256	0.47866	0.04627
RCC1221	2.92656	0.25378	PLY92A	0.61792	0.13032
RCC1254	2.49711	0.08247	RCC1222	0.94993	0.04210
RCC1208	0.71650	0.04868	BLOOM2195	0.60580	0.06661
RCC1248	0.63364	0.03153	RCC1258	0.81610	0.04466
RCC1251	2.14337	0.07085	CH24/90	1.35955	0.01822
RCC1710	1.69602	0.05115	5-9-25B	0.69739	0.01877
RCC1217	0.38351	0.01883	RCC1243	2.46560	0.24177
RCC1216	0.77526	0.06996			

Entries in bold denote strains displaying high activities.

**Table 11.** Principle Component scores (to 2 d.p.) for *E. huxleyi* strains in the enzyme activity screens.

**Strain**	**PC1**	**Strain**	**PC1**
RCC1812	−6.85	RCC1217	0.66
RCC1828	−6.44	RCC2054	0.78
RCC1269	−6.33	RCC1254	0.84
RCC1221	−5.33	RCC1268	0.91
CCMP373	−4.21	RCC1246	0.92
RCC1263	−3.81	RCC1258	1.03
CCMP2758	−2.53	5-9-25B	1.22
CCMP625	−2.47	N44-20D	1.39
RCC958	−2.21	RCC1818	1.46
CCMP2090inf	−1.96	RCC1250	1.76
CCMP376	−1.52	CCMP376-B	1.77
RCC1271	−1.15	CCMP2090	1.93
RCC1830	−1.10	RCC1256	1.94
RCC1251	−1.08	CCMP372	2.07
CCMP2758-B	−0.96	RCC962	2.15
RCC1270	−0.60	BLOOM2195	2.15
CCMP378	−0.55	CCMP374	2.18
RCC1710	−0.50	RCC1261	2.21
RCC1243	−0.40	CCMP88E	2.26
CH24/90	−0.07	RCC1850	2.26
RCC1257	−0.02	RCC1248	2.35
RCC1267	−0.01	RCC1208	2.46
RCC948	0.10	RCC912	2.47
CCMP1516	0.34	CCMP12.1	2.59
CCMP379	0.37	CCMP370	3.00
RCC1826	0.45	RCC1216	3.54
PLY92A	0.53		

Strains are arranged according to increasing PC1 score.
